# Self-mutilation of the Fifth Finger in an Infant due to Iatrogenic Ulnar Nerve Neurapraxia: A Clinical Case Report and Review of the Literature

**DOI:** 10.5435/JAAOSGlobal-D-19-00012

**Published:** 2019-08-02

**Authors:** Menahem Singer, Louis Schorr

**Affiliations:** From the Pediatric Orthopedics Unit, Wolfson Medical Center, Holon, Israel.

## Abstract

We describe a 1-year and 8-month-old girl who underwent closed reduction and percutaneous pinning by Kirschner wires of a fully displaced supracondylar humeral fracture. At follow-up, self-mutilation of the fifth finger was noticed. A nerve conduction study confirmed ulnar nerve neurapraxia, which allowed the patient to bite her fifth finger painlessly.

**Conclusion:**

Although self-mutilation after closed reduction and percutaneous pinning of supracondylar fractures was not previously described, and is probably very rare, a high index of suspicion and close follow-up is needed in infants in this setting.

Supracondylar fractures of the humerus are common pediatric elbow injuries. Ninety-eight percent of these fractures are extension type.^1^ Most displaced or angulated fractures are treated by closed reduction and percutaneous pinning.^2,3^ A meta-analysis that included 5148 patients showed that iatrogenic neuropathy occurred almost exclusively in the ulnar nerve (92.3%), because of injury by the medial pin, and was observed at an overall rate of 4.1%.^4^ To the best of our knowledge, no description is found in the literature of self-mutilation (autophagy) after nerve injury. Owing to the severe consequences of such an event and the simple prevention, we find it important to raise the awareness of neurapraxia in infants. The authors have obtained institutional review board exemption to report the case.

## Case Report

**Figure 1:**
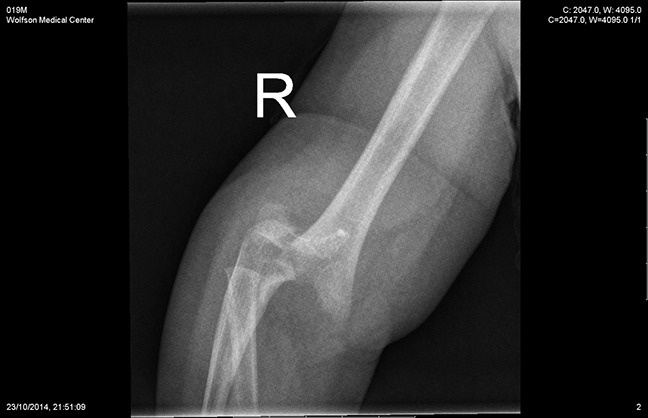
The initial radiograph of the fracture

**Figure 2:**
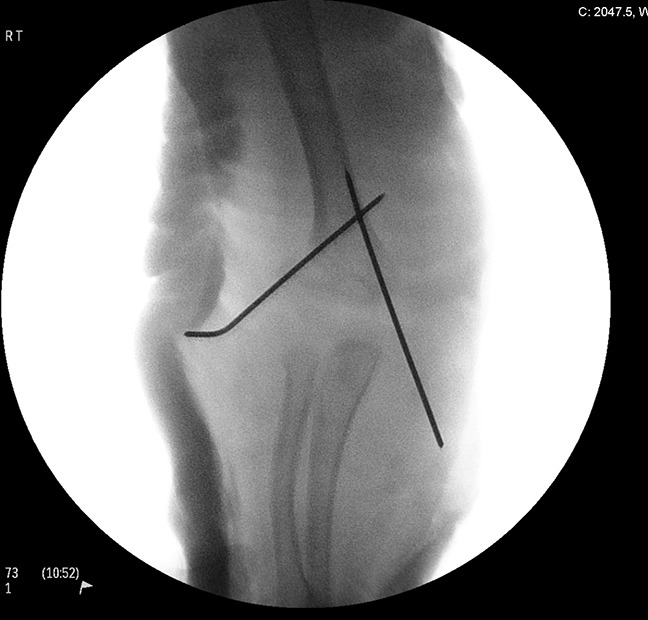
Radiograph showing immediate postsurgery position

A female patient aged 1 year 8 months old presented to the emergency department after falling on an outstretched hand. On radiograph imaging (Figure 1), a supracondylar fracture Gartland type III was diagnosed. On her first neurovascular survey, no abnormalities were noticed. The patient was operated on the next day. After closed reduction, two 1.4-mm Kirschner wires in a cross-configuration were placed to stabilize the fracture. On the lateral side, the wire was inserted percutaneously, and the medial pin was placed under direct bone vision. Good and stable fracture reduction was achieved (Figure 2). After surgery, the patient was placed in a posterior splint with 60° flexion at the elbow. A neurovascular examination was done, but no neurologic deficit (motor or sensory) was identified, although no documentation of specific sensation testing is found. Active finger movements were observed. The child was released from the hospital on the second day after surgery, with an outpatient clinic follow-up visit programmed 3 weeks after surgery.

**Figure 3:**
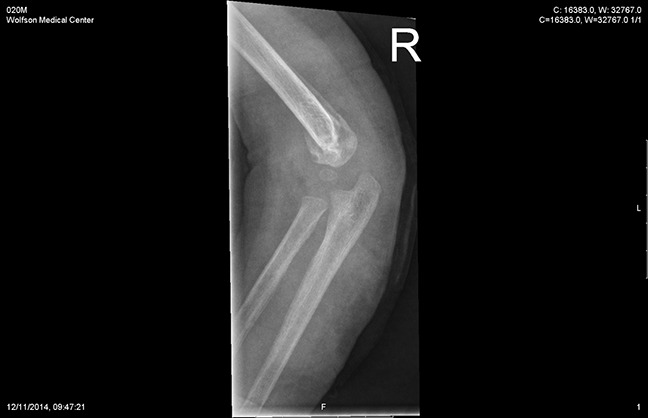
Radiograph after Kirschner wire removal

No motion was allowed at the elbow joint for all this period. The pins were removed after 3 weeks at the outpatient clinic visit (Figure 3).

**Figure 4:**
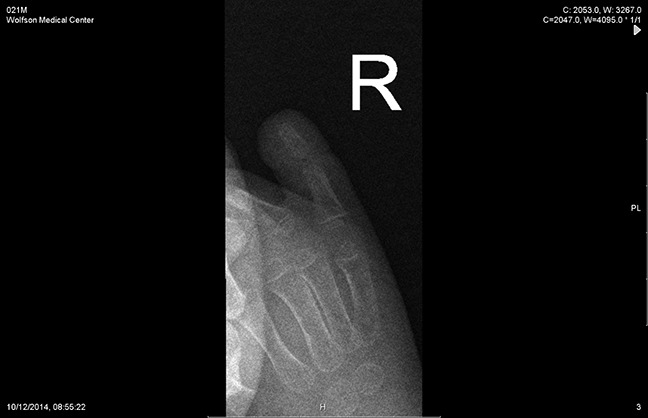
Radiograph of the distal phalanx amputation, oblique view

**Figure 5:**
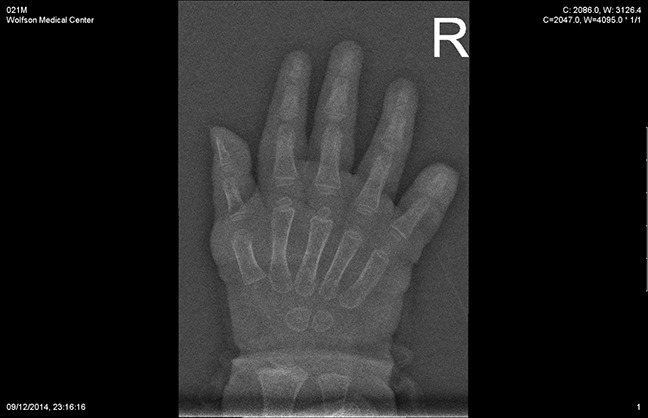
Radiograph of the distal phalanx amputation, AP view

About 6 weeks after surgery, the child began oral antibiotic treatment (Augmentin), which was prescribed by a physician in the community because of a skin infection on the fifth fingertip, on the fractured side. The mother saw the child bite her finger continuously. After a few days of treatment, the child was referred to the hospital because of lack of response to the antibiotic treatment. On her admission to the pediatric orthopaedics unit, amputation of the distal phalanx was noted, and radiograph imaging confirmed the diagnosis (Figures 4 and 5). A child abuse workup (total body examination and a social worker inquiry) that was done after admission ruled out this option. We started treatment with systemic intravenous antibiotics (Cefamezin 30 mg/kg) and topical treatment (Synthomycin).

On her second day in the hospital, a consultation was held with a pediatric neurologist to rule out a systemic disease. Our clinical suspicion was of an ulnar nerve injury sensory only without any motor deficit. Nerve conduction velocity (NCV) under light sedation was performed, which confirmed partial ulnar nerve injury.

The wound infection signs improved, and the wound began to heal under intravenous antibiotic treatment. An extra protection to the hand was done with a bulky bandage that the patient could not remove. The child was discharged from the hospital after 6 days of treatment, and she wore a mitten for about 1 month, 24 hours a day. She was kept under close clinical follow-up at the outpatient clinics once weekly. The wound healed. A second NCV was done after 3 months, and full nerve recovery was noticed. The patient did not bite her finger anymore.

At 1-year follow-up, the stump was not infected and was free of any tenderness or irritation. The child did not show any signs of ulnar nerve irritation.

## Discussion

A few descriptions of self-mutilation due to neuropathy have been described in the literature. Hereditary sensory and autonomic neuropathy type V is a rare inherited disease. Self-mutilation injury involving the teeth, lips, tongue, ears, eyes, nose, and fingers is an invariable feature of this disorder.^5^ A rare case of noncongenital nonpsychiatric autophagy of the fingers was published, and its cause was severe diabetic neuropathy.^6^

In our patient, the diagnosis of nerve injury was proposed only after a severe, unforeseen complication appeared. Only then did we perform NCV, which confirmed the clinical diagnosis.

In conclusion, identification of nerve injury in surgically treated infants is very difficult but nonetheless important. Orthopaedic surgeons should recognize that insensate extremities in young children are at risk of “self-mutilation” or even “autophagy”. Observation of self-mutilating behavior by infants should immediately be evaluated for neuropathy and treated by measures to prevent further self-mutilation such as irremovable protective mittens.

A high index of suspicion and close cooperation with parents are needed to reach accurate diagnosis in challenging cases such as the one described.
